# Supplementary material for: Global hypo-methylation in a proportion of glioblastoma enriched for an astrocytic signature is associated with increased invasion and altered immune landscape
Source: eLife. 2022 Nov 22;11:e77335. doi: 10.7554/eLife.77335 (PMC9681209; doi:10.7554/eLife.77335)
Supplement: Figure 2—source data 1. [file elife-77335-fig2-data1.zip › Figure_2_source_data_1/Figure_2A/homerResults/motif42.similar.html]

motif42

## Information for motif42

A
C
T
G
A
G
T
C
A
C
T
G
A
G
T
C
A
T
G
C
A
C
G
T
A
C
T
G
C
G
T
A
  
Reverse Opposite:  

A
C
G
T
G
T
A
C
G
T
C
A
A
T
C
G
C
T
A
G
A
G
T
C
A
C
T
G
A
G
T
C
  

|  |  |
| --- | --- |
| p-value: | 1e-13 |
| log p-value: | -3.217e+01 |
| Information Content per bp: | 1.927 |
| Number of Target Sequences with motif | 14.0 |
| Percentage of Target Sequences with motif | 0.60% |
| Number of Background Sequences with motif | 1.7 |
| Percentage of Background Sequences with motif | 0.05% |
| Average Position of motif in Targets | 82.5 +/- 53.2bp |
| Average Position of motif in Background | 111.6 +/- 25.6bp |
| Strand Bias (log2 ratio + to - strand density) | 0.8 |
| Multiplicity (# of sites on avg that occur together) | 1.00 |
| Motif File: | file (matrix) reverse opposite |

### Similar de novo motifs found

|  |  |  |  |  |  |  |  |
| --- | --- | --- | --- | --- | --- | --- | --- |
| Rank | Match Score | Redundant Motif | P-value | log P-value | % of Targets | % of Background | Motif file |
| 1 | 0.633 | T G A C T C A G A T C G C G A T A C T G A G T C G A T C C G A T C T G A C G T A | 1e-13 | -32.040191 | 1.46% | 0.29% | motif file (matrix) |
| 2 | 0.655 | C T G A C T A G C A T G C T A G A T C G G T A C G A C T A T G C G T A C G C A T T A C G T C G A | 1e-11 | -25.537087 | 0.65% | 0.07% | motif file (matrix) |
